# Relations of Menstruation, Lactation, and Conception

**Published:** 1853-05

**Authors:** 


*Relations of Menstruation, Lactation, and Conception.*—Dr. Robert Barnes, in a paper on this subject, in the February No. of the London Lancet, arrives at the following conclusions, based on the analysis of the histories of 100 women:

1. Lactation exercises a considerable influence in preventing menstruation and conception.
2. This influence appears to be marked and constant in some women, and to exist but feebly in others.
3. The influence of lactation in averting menstruation or conception, cannot, for the most part, be kept up longer than twelve months.
4. There is a close relation between the occurrence of menstruation, during suckling, and conception; that is, when menstruation appears during suckling, conception is very likely to follow.
5. When pregnancy takes place during suckling, and suckling is continued, abortion is very apt to follow.
6. The chief causes of the abortions brought about during suckling, are the revolt of the ovaria and uterus, evinced by the return of the menstrual nisus, and the deterioration of the mother's blood; to which must be added, superinduced disease of the ovum.
7. The practical conclusion that weaning should be enjoined, not only whensoever pregnancy takes place, but also whensoever menstruation returns.

